# The transcription factor KLF14 regulates macrophage glycolysis and immune function by inhibiting HK2 in sepsis

**DOI:** 10.1038/s41423-021-00806-5

**Published:** 2022-01-04

**Authors:** Yuan Yuan, Guangjian Fan, Yuqi Liu, Lu Liu, Tong Zhang, Pengfei Liu, Qing Tu, Xinyi Zhang, Shiyuan Luo, Liangfang Yao, Feng Chen, Jingbao Li

**Affiliations:** 1 Department of Anesthesiology, Shanghai General Hospital, Shanghai Jiao Tong University, Shanghai, 201620 China; 2 Precision Research Center for Refractory Diseases, Shanghai General Hospital, Shanghai Jiao Tong University, Shanghai, 201620 China; 3 Department of Anesthesiology, Weifang Medical University, Weifang, 261000 China; 4 Department of Anesthesiology, Ruijin Hospital, Shanghai Jiao Tong University School of Medicine, Shanghai, 201620 China

**Keywords:** Sepsis, Macrophages, KLF14, HK2, Glycolysis, Perhexiline, Monocytes and macrophages, Sepsis

## Abstract

Sepsis is a heterogeneous syndrome induced by a dysregulated host response to infection. Glycolysis plays a role in maintaining the immune function of macrophages, which is crucial for severely septic patients. However, how the pathways that link glycolysis and macrophages are regulated is still largely unknown. Here, we provide evidence to support the function of KLF14, a novel Krüppel-like transcription factor, in the regulation of glycolysis and the immune function of macrophages during sepsis. KLF14 deletion led to significantly increased mortality in lethal models of murine endotoxemia and sepsis. Mechanistically, KLF14 decreased glycolysis and the secretion of inflammatory cytokines by macrophages by inhibiting the transcription of HK2. In addition, we confirmed that the expression of KLF14 was upregulated in septic patients. Furthermore, pharmacological activation of KLF14 conferred protection against sepsis in mice. These findings uncover a key role of KLF14 in modulating the inflammatory signaling pathway and shed light on the development of KLF14-targeted therapeutics for sepsis.

## Introduction

Sepsis is the leading cause of death worldwide and is defined as life-threatening multiple organ dysfunction caused by dysregulation of the host’s response to infection [1]. During pathogen invasion, the innate immune response reacts as the first barrier to defend the host and initiates the inflammatory-immune reaction to remove pathogens. Moreover, persistent infection leads to an immune response disorder associated with hyperinflammation, which is characterized by an excessive release of proinflammatory cytokines known as a cytokine storm, leading to organ dysfunction or failure and even death [2]. Mononuclear macrophages are a bridge between innate immunity and adaptive immunity. In the early stage of sepsis, activated mononuclear macrophages phagocytose pathogens, perform antigen-presentation functions, and secrete proinflammatory cytokines. As sepsis progresses to the immunosuppressive stage, the immune functions of macrophages, such as phagocytosis, antigen presentation, and secretion of proinflammatory factors, are inhibited [3]. Studies have shown that the functional state of mononuclear macrophages is closely related to the mortality of patients with sepsis [4]. Recent findings have shown that macrophage functions are closely related to glycolysis, and adjusting the level of glycolysis can improve the immune function of macrophages [5]. Therefore, finding the crucial regulatory metabolic nodes that control immune cell function has become a focus of research in the field of sepsis treatment [6].

Krüppel-like transcription factors (KLFs) have multiple biological regulatory functions and participate in many crucial pathological processes, including cell proliferation, differentiation, and apoptosis [7]. Findings have shown that KLFs are widely distributed in the cardiovascular, respiratory, gastrointestinal, and immune systems [8, 9]. KLF14 is a member of the SP/KLF family that contains a zinc-finger structure at its C-terminus and is encoded on human chromosome 7. Moreover, KLF14 is a maternally expressed gene and has a large CpG island across most regions of the open reading frame (ORF). This CG-rich region regulates downstream gene expression and nuclear protein transcription by interacting with coactivators or immune complexes [10]. Previous studies have shown that KLF14 significantly regulates lipid metabolism and blood glucose levels, which are closely related to the pathogenesis of atherosclerosis, type 2 diabetes, insulin resistance, and other diseases [11]. A recent study further implied that KLF14 participates in the differentiation of Treg cells by binding to a specific demethylation enhancer of Treg cells [12]. Notably, additional studies have identified KLF14 as a tumor suppressor in the development and progression of colorectal cancer [13]. Moreover, KLF14 was also found to regulate cell signaling pathways and immune function [14]. While increasing evidence has confirmed the impacts of KLF14 on immunity and metabolism, whether KLF14 can affect the immune function of macrophages through glycolysis has not been reported thus far.

All these facts suggest that KLF14 may play a role in inflammatory disease. We sought to further understand the role of KLF14 in the regulation of the inflammatory response in sepsis, and we first observed the increased expression of KLF14 in sepsis patients and mice. Then, we explored the functional effects in vitro and elucidated the mechanism of KLF14 in the immune functions of macrophages in sepsis. These experiments provide an additional explanation for sepsis-induced immune dysfunction. Here, our data indicate that the KLF14-HK2 signaling pathway can affect the immune function of macrophages, suggesting that KLF14 is a potential therapeutic target for sepsis.

## Methods

### Patient samples

Peripheral blood mononuclear cells (PBMCs) from sepsis patients (*n* = 7) and healthy controls (*n* = 8) were collected from Shanghai General Hospital between January 2019 and December 2020 (for patient characteristics, see Supplementary Table 1). Sepsis was identified according to the Third International Consensus Definitions for Sepsis and Septic Shock (Sepsis-3) [1]. This study was approved by the Ethics Committee of Shanghai General Hospital (ethical approval number: 2019KY033).

### Animal models of sepsis

C57BL/6 wild-type mice were purchased from Shanghai SLAC Laboratory Animal Co. KLF14^−/−^ mice were donated by Professor Wang Chuangui (Clinical Transformation Research Institute of Shanghai General Hospital). The genotyping of KLF14 knockout mice was performed as previously described [15]. The sequences of the primers used to identify mouse genotypes were 5’-CGCCGTGGCTTGCCTGGAC-3’ and 5’ -TTCGGGGTCCGTTGCGCG-3’. Mice were housed in groups of five animals per cage in a temperature-controlled facility and kept on a regular 12-hour light and dark cycle. Mice were maintained and experimental procedures were performed under pathogen-free conditions. In this study, all procedures were carried out in accordance with the Guides For The Care And Use Of Laboratory Animals (National Academy of Sciences, China) and with approval from the Experimental Animal Management Ethics Committee of Shanghai Jiao Tong University School of Medicine (ethical approval number: 2019AW009).

#### Endotoxemia model

Male wild-type and KLF14^−/−^ knockout mouse and female wild-type and KLF14^−/−^ knockout mouse (6–8 weeks, 20–25 g) endotoxemia models were induced by intraperitoneal injection of LPS (20 mg/kg; Sigma, #L2630). Twelve hours after LPS injection, the mice were euthanized by an overdose of pentobarbital (100 mg/kg). Tissue samples were stored at −80 °C for subsequent analysis.

#### CLP model

Male wild-type and KLF14^−/−^ knockout mouse and female wild-type and KLF14^−/−^ knockout mouse (6–8 weeks, 20–25 g) CLP models were induced by cecal ligation and puncture by using a surgical procedure as previously described. Anesthesia was induced with sevoflurane (2–4%, inhalation). The cecum was exteriorized from a small midline abdominal incision and ligated with 4-0 silk. Then, a 25-gauge syringe needle was used to puncture the cecum. The peritoneum and skin were sutured in turn, and the mice were injected subcutaneously with 1 ml of saline solution. After 12 h of CLP, mice were euthanized by an overdose of pentobarbital (100 mg/kg). Tissue samples were stored at −80 °C for subsequent analysis.

### Cell culture

The HEK-293T, RAW264.7, and THP-1 cell lines were purchased from the Cell Bank of the Chinese Academy of Sciences. The 293T and RAW264.7 cell lines were cultured in DMEM with 10% fetal bovine serum (FBS) and 1× penicillin-streptomycin. THP-1-derived macrophages were cultured in 1640 medium with 10% FBS, 1× penicillin-streptomycin and 0.05 mM β-mercaptoethanol and treated with PMA (100 ng/ml; #P1585, Sigma) for 72 h. Cells were routinely cultured in a CO_2_ incubator at 37 °C.

According to the protocols for Ficoll density gradient centrifugation, PBMCs were collected from human blood samples by using lymphocyte separation medium. Bone marrow-derived macrophages (BMDMs) were harvested from the femurs and tibias of wild-type or KLF14^−/−^ mice. The suspension was washed with PBS and cultured in DMEM with 10% fetal bovine serum (Gibco), 1× penicillin-streptomycin, and M-CSF (10 ng/ml; #100-21-10, PeproTech). BMDMs were dispensed into 6-well culture plates and stimulated with LPS (100 ng/ml, 6 h; Sigma, #L5293) after culture for 7 days.

### Western blot

The cells and tissues were washed with 1× PBS and lysed in RIPA lysis buffer containing protease inhibitors. The cell lysates were separated by SDS-PAGE, transferred to PVDF membranes, and immunoblotted with the indicated antibodies. The antibodies used were as follows: KLF14 (1:1000; #23784, Invitrogen); HK2 (1:1000; #22039, Proteintech); α-tubulin (1:2500; #11224, Proteintech); IL-1β (1:1000; #12242S, CST); Flag (1:2000; #20543, Proteintech); and Flag (for the ChIP experiment; #14793S, CST).

### Quantitative real-time PCR

Total RNA was extracted from samples using TRIzol reagent (#9109, TAKARA), and 1 μg of RNA was reverse transcribed to generate cDNA (#R323-01-AC, Vazyme). The cDNA was subjected to qRT-PCR by using SYBR Green Master Mix (#Q711-02-AA, Vazyme). The primer sequences are listed in Table 1.

**Table 1:** Sequences of the primers used for this study

Gene	Forward sequence 5’−3’	Reverse sequence 5’−3’
KLF14	CCTCAAGTCACACCAGCGTA	CGACCTCGGTACTCGATCAT
HK2	TGATCGCCTGCTTATTCACGG	AACCGCCTAGAAATCTCCAGA
ENO1	TGCGTCCACTGGCATCTAC	CAGAGCAGGCGCAATAGTTTTA
IL-1β	CTGTGACTCATGGGATGATGATG	CGGAGCCTGTAGTGCAGTTG
IL-6	CCAAGAGGTGAGTGCTTCCC	CCAAGAGGTGAGTGCTTCCC
ALDOB	GAAACCGCCTGCAAAGGATAA	GAGGGTCTCGTGGAAAAGGAT
LDHB	CATTGCGTCCGTTGCAGATG	GGAGGAACAAGCTCCCGTG
β-Actin	GGCTGTATTCCCCTCCATCG	CCAGTTGGTAACAATGCCATGT

### Cell transfection

The KLF14 and HK2 overexpression plasmids were both cloned into the pCDNA3.1 vector with a Flag tag. The KLF14 siRNA sequences were 5’-GCUGCACCAAAGCCUAUUATT-3’ and 5’ -UAAUAGGCUUUGGUGCAGCTT-3’. 293T and RAW264.7 cells were transfected with small interfering RNA to knock down the expression of KLF14 using Lipofectamine 2000 reagent (#11668027, Invitrogen). 293T and RAW264.7 cells were transfected with KLF14 overexpression plasmids using EZ trans reagents (#AC04L011, Shanghai Life iLab Bio-Technology). All cells were stimulated with LPS after transfection for 48 h.

### Biochemical analysis

The levels of IL-1β were measured using an ELISA kit (Cat No. MLB00C, R&D). The levels of IL-6 were measured using an ELISA kit (Cat No. M6000B, R&D). The levels of TNF-α were measured using an ELISA kit (Cat No. MTA00B, R&D). Extracellular lactate levels were determined using a lactate assay kit (Cat No. MAK064, Sigma–Aldrich).

### Glucose uptake

Glucose uptake was detected using a glucose uptake assay (Cat No. ab234043, Abcam). On Day 7 of culture, macrophages were seeded into 24-well plates at a density of 5 × 10^5^ cells per well overnight. Cells were treated with or without 100 ng/ml LPS for 6 h before starvation, and then the culture medium was replaced with glucose-free medium without serum for 2 h. According to the manufacturer’s protocol, cells were treated with glucose and hexokinase inhibitors and incubated at 37 °C with 5% CO_2_ for 30 min. The cells were washed twice with ice-cold 1× PBS, lysed for 10 min, and centrifuged at 12,000 × *g* for 5 min. Sample supernatant (20 μl) was added to a 96-well plate with reaction mix for glucose uptake measurement using a fluorescence microplate reader at Ex/Em = 535/587.

### ECAR and OCR

After culture for 7 days, macrophages were seeded into XFe96 microplates at a density of 1 × 10^5^ cells per well and cultured overnight. Cells were treated with or without 100 ng/ml LPS for 6 h before extracellular flux analysis was performed. The culture medium was switched to XF DMEM base medium (Cat No. 103575-100, Agilent) supplemented with glucose (10 mM), pyruvate (1 mM), and glutamine (2 mM). Cells were incubated in a non-CO_2_ incubator at 37 °C for 1 h. The extracellular acidification rate (ECAR) and oxygen consumption rate (OCR) were measured using an XFe96 analyzer after sequential injection of the compounds of the XF Glycolysis Stress Test Kit (Cat No. 103020-100, Agilent) (rotenone and antimycin A (0.5 μM) and 2-deoxyglucose (50 mM)) or the XF Cell Mito Stress Test Kit (Cat No. 103015-100, Agilent) (oligomycin (1.5 µM), FCCP (2 µM), and rotenone and antimycin A (0.5 µM)). The ECAR and OCR were automatically calculated by Seahorse XFe96 software (Seahorse Bioscience, Agilent).

### Luciferase reporters

293T cells (5 × 10^4^/well) were cultured on a 24-well plate and cotransfected with overexpression plasmids for KLF14 or a KLF14 zinc-finger 2 point mutation plasmid (ΔZF2) and plasmids containing human HK2 promoter fragments (pGL3-HK2-Luc) by using EZ trans reagents (Cat No. AC04L011, Shanghai Life iLab Bio-Technology). Cells were cultured for 24 h after transfection, and the luciferase activity was measured with the Dual-Luciferase Reporter Assay System Kit (Cat No. DL101-21, Vazyme).

### Murine bone marrow transplant

C57BL/6 WT mice were used as recipients to conduct a bone marrow transplantation experiment after lethal total-body irradiation (9.5 Gy). The day after irradiation, 1 × 10^6^ bone marrow cells from WT C57BL/6 mice or KLF14^−/−^ mice were transplanted into C57BL/6 WT mice by intravenous injection and treated with antibiotics. Eight weeks after transplantation, murine endotoxemia and sepsis models were established.

### Chromatin immunoprecipitation (ChIP)

293T cells were transfected with KLF14-Flag or vector plasmid for 48 h, and then ChIP assays were performed using the CUT & RUN Kit (#86652, Cell Signaling Technology). Following the protocol, the resulting DNA products were quantified by q-PCR. The sequences of the primers for the HK2 promoter were 5’-CCCATAGCCGAGCCTGACCTGGAC-3’ and 5’ -CGCATGAGCCACCGCCGC and 5’-CTGAGATGGGACGTGTGGT-3’ and 5’ -CGTCCCAGCCTTTAGCCACGG-3’.

### Statistical analysis

Results were expressed as the mean ± SD. GraphPad Prism 8 was used for statistical analysis. Unpaired two-tailed Student’s *t* tests were used to compare two samples. One-way ANOVA was used for comparisons among the different groups. Kaplan–Meier analysis was used to compare differences in survival rates between groups. Differences were considered significant at a *P* value <0.05.

## Results

### KLF14 is upregulated in macrophages in in vivo and in vitro sepsis models

**Fig. 1:**
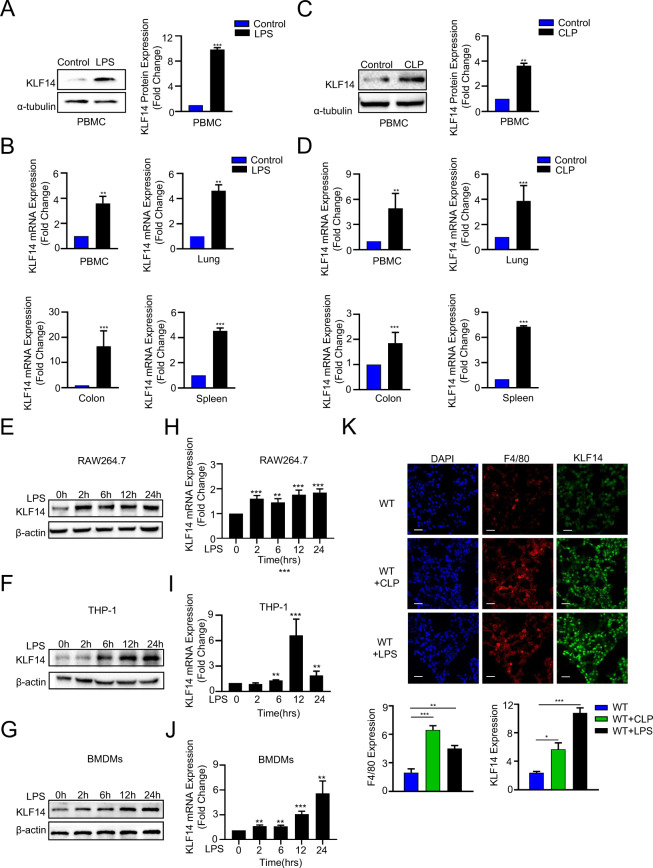
KLF14 is upregulated in the macrophages of in vivo and in vitro murine sepsis models. **A**–**D** Peripheral mononuclear macrophages (PBMCs), lung tissues, colon tissues and spleen tissues were isolated from endotoxemic mice (*n* = 3; LPS 20 mg/kg ip, 12 h), septic mice (*n* = 3; CLP 25-gauge needle, 12 h) and healthy controls (*n* = 3). **A**, **C** The expression of the KLF14 protein in PBMCs from endotoxemic, septic, and healthy mice. **B**, **D** The mRNA expression of KLF14 in PBMCs and lung, colon, and spleen tissues from endotoxemic, septic, and healthy mice. The data are presented as the mean ± SD. *n* = 3, **P < 0.01, ***P < 0.001. **E**–**J** RAW264.7 macrophages, THP-1-derived macrophages, and BMDMs were treated for 0 h, 2 h, 6 h, 12 h, and 24 h with LPS (100 ng/ml). Protein (**E**–**G**) and mRNA (**H**–**J**) expression of KLF14 in RAW264.7 macrophages, THP-1-derived macrophages, and BMDMs. The data are presented as the mean ± SD. *n* = 3, **P < 0.01, ***P < 0.001. **K** Immunofluorescence staining of the macrophage markers F4/80 (red) and KLF14 (green) and DAPI (blue) in lung sections taken 12 h after intraperitoneal LPS injection or cecal ligation puncture. Scale bar, 20 µm. The data are presented as the mean ± SD. *n* = 3, *P < 0.05, **P < 0.01, ***P < 0.001

Previous observations have demonstrated that KLF14 is involved in T cell differentiation and that a lack of KLF14 results in protection against colitis, indicating that KLF14 is involved in the regulation of the immune response. To determine the role of KLF14 in the immune function of macrophages during sepsis, we first assessed the expression of KLF14 in in vivo and in vitro murine sepsis models (Fig. 1). In lethal models of murine sepsis and endotoxemia, the expression of KLF14 was increased in PBMCs and various tissues of septic mice (Fig. 1A–D). Thus, the transcription factor KLF14 is involved in the process of sepsis.

We then investigated whether KLF14 was expressed in response to LPS stimulation in macrophages. The results showed that the expression of KLF14 increased in a time-dependent manner in RAW264.7 cells, THP-1-derived macrophages, and BMDMs in response to LPS stimulation (Fig. 1E–J) (Supplementary Fig. 1). Moreover, we also observed that the expression of KLF14 and F4/80 (a marker of macrophages) was upregulated in the lung tissues of septic mice (Fig. 1K). These results strongly suggest that KLF14 is involved in the inflammatory-immune regulation of macrophages during sepsis.

### KLF14 deletion can significantly induce severe inflammation in murine endotoxemia and sepsis models

**Fig. 2:**
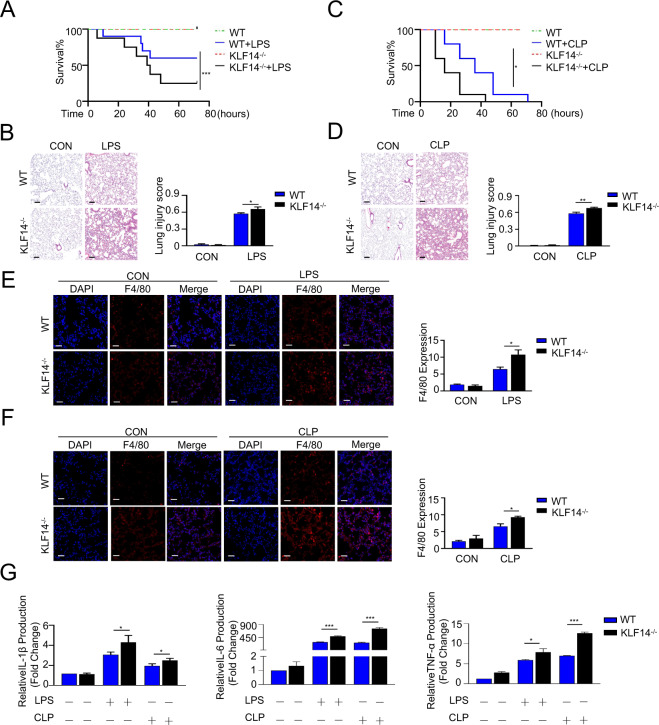
Deletion of KLF14 can significantly induce severe inflammation in murine endotoxemia and sepsis models. **A**, **C** The deletion of KLF14 in mice decreased the survival rate of mice with LPS (20 mg/kg)-induced endotoxemia and CLP (25-gauge needle)-induced sepsis (the data are presented as the mean ± SD, *n* = 15, mice per group; *P < 0.05, ***P < 0.001, Kaplan–Meier survival analysis). **B**, **D** Lung tissues of mice with CLP-induced sepsis and LPS-induced endotoxemia were stained with hematoxylin and eosin (12 h; scale bars, 100 µm). **E**, **F** Immunofluorescence staining of lung sections for the macrophage marker F4/80 (red) and DAPI (blue) (12 h; scale bar, 20 µm) and **G** ELISA analysis of cytokines in peripheral blood from mice with CLP-induced sepsis and LPS-induced endotoxemia (the data are presented as the mean ± SD, *n* = 3, *P < 0.05, ***P < 0.001)

To understand the impact of KLF14 deletion on the biological responses of septic mice, we used KLF14 knockout mice (Supplementary Fig. 2). Male wild-type (WT) and KLF14^−/−^ mice were subjected to endotoxemia induced by intraperitoneal injection and sepsis induced by cecal ligation and puncture. Notably, the survival rate of KLF14^−/−^ septic mice was significantly decreased compared with that of WT septic mice (Fig. 2A, C). We assessed lung tissue injury to evaluate the severity of the tissue inflammatory response in septic mice. The results showed an increase in leukocyte infiltration and alveolar septal wall thickening in the lungs of endotoxemic and septic mice. The lung injury score in KLF14^−/−^ mice was substantially higher than that in WT mice (Fig. 2B, D). To further elucidate the possible role of KLF14 in macrophages during sepsis, we assessed the infiltration of macrophages into the septic and normal lungs of WT and KLF14^−/−^ mice. The results showed that the number of infiltrated macrophages in the lungs of septic mice was higher than that in the lungs of control mice, and the F4/80 fluorescence intensity in KLF14^−/−^ septic lungs was significantly higher than that in WT septic lungs (Fig. 2E, F). We detected the serum levels of inflammatory cytokines in the peripheral blood of septic and control mice. The secretion of proinflammatory cytokines, including IL-1β, IL-6, and TNF-α, in KLF14^−/−^ septic mice was strikingly higher than that in WT septic mice (Fig. 2G). Moreover, it has been reported that KLF14 is a maternally expressed gene; therefore, we also established female murine sepsis models, and the results showed that the deletion of KLF14 also increased the inflammation level in female sepsis mice (Supplementary Fig. 3). These findings indicate that KLF14^−/−^ murine sepsis models display an increased inflammatory response.

### KLF14 plays a crucial role in the immune response of macrophages during sepsis

Previous results have shown that the deletion of KLF14 can lead to more macrophage infiltration in the lung tissues of septic mice (Fig. 2E, F). To further understand the effect of KLF14 on the macrophage immune response during sepsis, we performed transplant experiments by using WT C57BL/6 mice as recipients. Bone marrow cells from WT C57BL/6 mice or KLF14^−/−^ mice were transplanted into WT C57BL/6 mice to achieve the goal of KLF14 deletion in BMDMs (Supplementary Fig. 4A). Eight weeks after transplantation, we established murine endotoxemia and sepsis models. We observed injury to lung tissue by HE staining and q-PCR experiments (Supplementary Fig. 4B–E). The results showed that the WT C57BL/6 mice receiving KLF14^−/−^ bone marrow cell transplantation had a more severe inflammatory response in the lung tissues during sepsis, and the ELISA results of the serum levels of inflammatory cytokines in the peripheral blood of septic and control mice were consistent (Supplementary Fig. 4F–G).

**Fig. 3:**
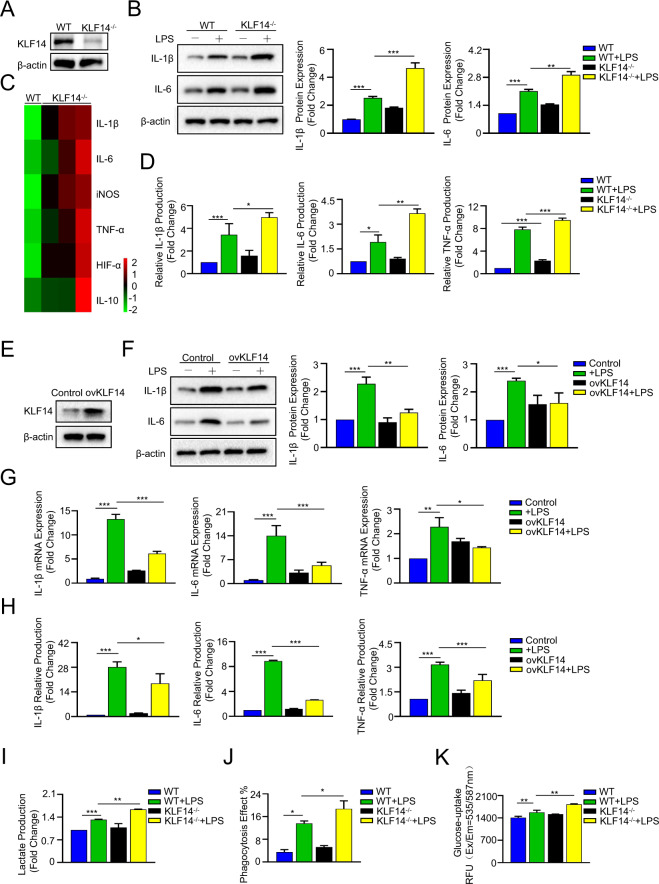
KLF14 plays a crucial role in the immune response of macrophages during sepsis. **A** Western blot analysis of the indicated protein expression in BMDMs confirmed the deletion of KLF14. **B** The protein expression of IL-1β and IL-6 in BMDMs after stimulation with LPS (100 ng/ml) for 6 h. **C** Heatmap of inflammatory cytokine changes in WT and KLF14^−/−^ BMDMs. **D** ELISA analysis of cytokines secreted from BMDMs after LPS (100 ng/ml) stimulation for 6 h. **E** Western blot analysis of the indicated protein expression in RAW264.7 macrophages confirmed the overexpression of KLF14. The indicated protein expression (**F**), mRNA expression (**G**), and ELISA results (**H**) of inflammatory cytokines in the control group and KLF14 overexpression group after LPS (100 ng/ml) stimulation for 6 h were assayed. The data are presented as the mean ± SD. *n* = 3, *P < 0.05, **P < 0.01, ***P < 0.001. **I** The lactate production, **J** phagocytosis, and **K** glucose uptake of WT and KLF14^−/−^ BMDMs stimulated with or without 100 ng/ml LPS for 6 h were assayed. The data are presented as the mean ± SD. *n* = 3, *P < 0.05, **P < 0.01, ***P < 0.001

In an in vitro study, we cultured BMDMs from WT and KLF14^−/−^ mice (Fig. 3A). We found that the KLF14^−/−^ BMDMs were more responsive to LPS stimulation, with higher expression of IL-1β and IL-6 than that observed in the WT groups (Fig. 3B). Moreover, we also evaluated inflammation levels by q-PCR and ELISA. Our results showed that both the mRNA level and the secretion level of inflammatory cytokines of KLF14^−/−^ BMDMs were markedly higher than those of WT BMDMs (Fig. 3C, D). These data indicate that KLF14 deficiency exacerbates the immune response to LPS stimulation in BMDMs.

In addition, we further observed the regulatory effect of KLF14 in RAW264.7 cells. After transfection with KLF14 plasmids (Fig. 3E) (Supplementary Fig. 5), the results showed that compared with the control, the mRNA and protein levels of inflammatory cytokines, including IL-1β, IL-6 and TNF-α, were decreased in KLF14-overexpressing RAW264.7 cells stimulated with LPS (Fig. 3F, G). Moreover, the cytokine secretion quantified by ELISA confirmed that overexpression of KLF14 could also reduce the secretion of IL-1β, IL-6, and TNF-α in LPS-stimulated macrophages (Fig. 3H). These results further confirmed the regulatory functions of KLF14 in the immune response of macrophages. Moreover, our results also showed that the deletion of KLF14 affected lactate acid secretion, phagocytosis, and glucose uptake (Fig. 3I–K) in macrophages upon stimulation with LPS. These results indicate that KLF14 plays a crucial role in the immune response of macrophages during sepsis.

### KLF14 regulates glycolytic activity in macrophages

**Fig. 4:**
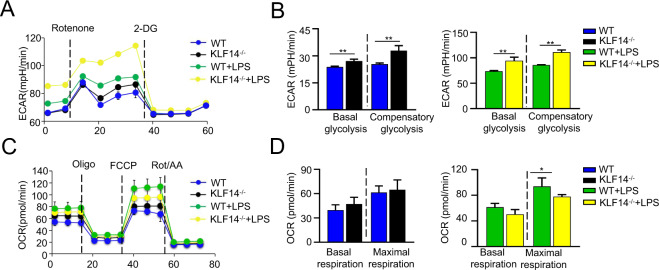
KLF14 regulates glycolytic activity in macrophages. **A**, **B** The ECAR of BMDMs isolated from WT and KLF14^−/−^ mice stimulated with or without 100 ng/ml LPS for 6 h was assessed by the Seahorse assay before and after sequential addition of rotenone and 2DG. The data are presented as the mean ± SD. *n* = 3, **P < 0.01. **C**, **D** The OCR was assessed by the Seahorse assay before and after sequential addition of oligomycin, FCCP, rotenone, and antimycin. The data are presented as the mean ± SD. *n* = 3, *P < 0.05

Macrophages switch their metabolism from oxidative phosphorylation to glycolysis when exposed to an external stimulus to generate ATP more quickly. It has been suggested that the transcription factor KLF14 is involved in metabolic diseases [11] and regulates glucose metabolism through the PI3K/Akt signaling pathways [16]. Our studies have identified the regulatory role of KLF14 in the immune response of macrophages during sepsis. To further understand whether KLF14 can affect immune responses to sepsis by regulating macrophage glycan metabolism (Fig. 4), we assessed two main modes of metabolism in macrophages: glycolysis and mitochondrial metabolism. We measured glycolytic and mitochondrial metabolic activity using a Seahorse XFe96 analyzer. Our results showed that the level of glycolysis in KLF14^−/−^ BMDMs was markedly higher than that in WT BMDMs with or without LPS stimulation (Fig. 4A, B). However, the level of mitochondrial metabolism in KLF14^−/−^ BMDMs was not substantially different from that in the WT groups (Fig. 4C, D). After LPS stimulation, maximal respiration and ATP production were reduced in KLF14^−/−^ BMDMs compared with WT BMDMs, indicating that KLF14 mainly modulates the glycolytic function of macrophages in response to LPS. Together, these findings suggest that KLF14 may act as a key modulator of the immune response of macrophages by affecting glycolysis during sepsis.

### KLF14 modulates glycolysis in macrophages by inhibiting the transcription of hexokinase 2

**Fig. 5:**
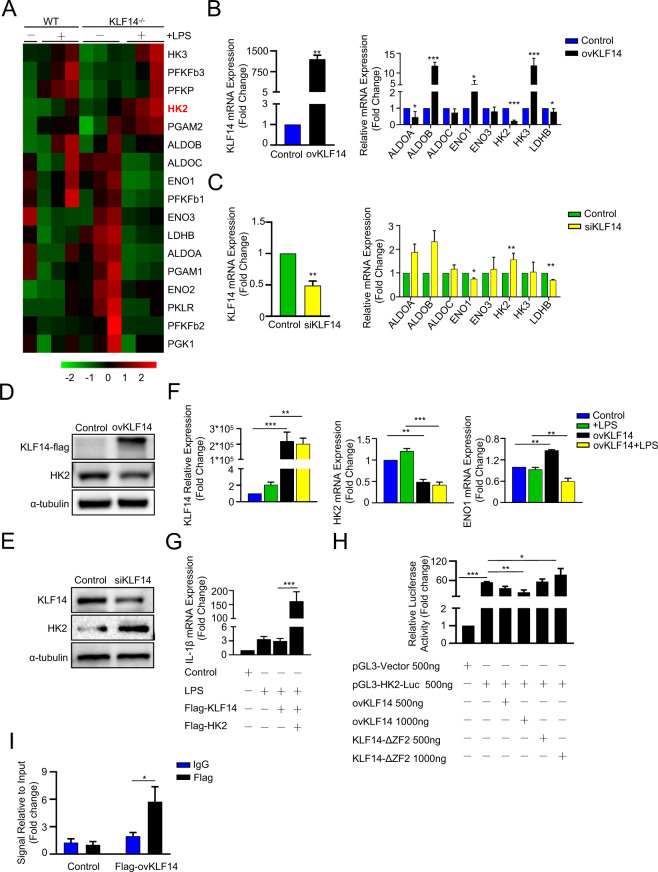
KLF14 modulates glycolysis in macrophages by inhibiting the transcription of hexokinase 2. **A** Heatmap of glycolysis-related enzyme changes in WT and KLF14^−/−^ BMDMs stimulated with LPS or not (100 ng/ml). 293T cells were transfected with KLF14-Flag plasmids or KLF14 siRNA, **B**, **C** the mRNA expression of KLF14 and glycolysis enzymes, and **D**–**E** Western blot analysis of the indicated protein expression was performed. The data are presented as the mean ± SD, *n* = 3, *P < 0.05, **P < 0.01, ***P < 0.001. **F** The mRNA expression of KLF14, HK2, and ENO1 in RAW264.7 macrophages after upregulating the expression of KLF14 with LPS stimulation (100 ng/ml) for 6 h. **G** Transfection of the HK2 overexpression plasmid alleviated the mRNA expression of IL-1β inhibited by KLF14. **H** Human HK2 promoter luciferase reporter (pGL3-HK2-Luc) was cotransfected with Flag-tagged KLF14 or KLF14-ΔZF2 (zinc finger 2 point mutation) plasmids in 293T cells for 24 h, and then relative luciferase activity was analyzed. **I** ChIP assay was conducted in 293T cells overexpressing KLF14-flag using anti-Flag antibody and anti-IgG antibody and analyzed by q-PCR using two pairs of primers of the HK2 promoter. The data are presented as the mean ± SD, n = 3, **P < 0.01, ***P < 0.001

To investigate the targets via which KLF14 regulates glycolysis in macrophages, we assessed the mRNA expression of glycolytic enzymes in macrophages. The results showed that the expression of some of the glycolytic enzymes in KLF14^−/−^ BMDMs was significantly increased compared with that in WT BMDMs (Fig. 5A). We observed that the key glycolytic enzyme HK2 (hexokinase 2) was significantly increased in KLF14^−/−^ BMDMs compared with WT BMDMs in response to LPS stimulation (Fig. 5A). Next, we used gain-of-function and loss-of-function studies to further confirm the relationship between HK2 and KLF14 in the immune response of macrophages (Fig. 5B–E). We constructed a KLF14 overexpression plasmid to increase the expression of KLF14 in 293T cells. We found that the mRNA expression of several glycolytic enzymes was upregulated in 293T cells after transfection. In contrast, the expression of other enzymes, such as ALDOA (aldolase, fructose-bisphosphate A), ALDOC (aldolase, fructose-bisphosphate C), HK2, and LDHB (lactate dehydrogenase B), was downregulated (Fig. 5B). Then, siRNA was used to inhibit the expression of KLF14, and the results showed that only the expression trend of the glycolytic enzyme HK2 was consistent with the previous results, with HK2 expression showing an inverse relationship with KLF14 expression (Fig. 5C). Thus, the data suggest that HK2 is highly likely to be a target of KLF14 involved in the glycolytic function of macrophages, and we verified this idea at the protein level (Fig. 5D, E) (Supplementary Fig. 6). To further clarify whether KLF14 regulates HK2 expression in macrophages, we overexpressed KLF14 in RAW264.7 cells. Consistently, compared with that of ENO1, the expression of HK2 was always decreased when KLF14 was upregulated in RAW264.7 cells with or without LPS stimulation (Fig. 5F). Furthermore, we performed rescue assays to identify the role of HK2 and KLF14 in the immune response of macrophages. Consistent with the previous results, we first observed that the mRNA expression of IL-1β decreased after the upregulation of KLF14 with LPS stimulation in RAW264.7 cells. Subsequently, we found that after transfection of the HK2 overexpression plasmid, the decrease in the mRNA expression of IL-1β inhibited by KLF14 was reversed (Fig. 5G). Previous findings have indicated that KLF14 induces transcriptional regulation by binding to promoters. Here, we constructed a luciferase reporter containing a segment of the human HK2 promoter to examine the effect of KLF14 on promoter activity. In addition, we investigated the transcriptional regulation of HK2 by using a Flag-tagged KLF14 plasmid or a KLF14 zinc-finger 2 site mutation plasmid (KLF14-ΔZF2) [15]. As it carries a point mutation affecting the KLF14 zinc-finger 2 structure, the KLF14-ΔZF2 plasmid had no transcriptional regulation function (Fig. 5H). The results showed that the KLF14 overexpression plasmid reduced the promoter activity of HK2 in a dose-dependent manner, but the KLF14-ΔZF2 plasmid did not, indicating that KLF14 regulates transcription by binding to the HK2 promoter. In addition, we performed ChIP assays in 293T cells by using anti-Flag and anti-IgG antibodies and q-PCR analysis of the genomic fragment using a pair of primers for the HK2 promoter. This result revealed recruitment of KLF14 to the HK2 promoter (Fig. 5I). Collectively, these data indicated the transcriptional regulation of HK2 by KLF14.

### KLF14 is upregulated in sepsis patients

**Fig. 6:**
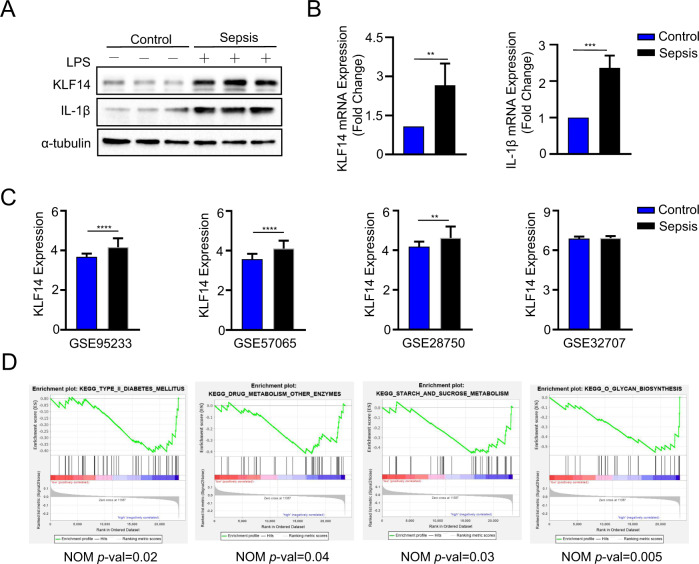
KLF14 is upregulated in sepsis patients. Peripheral mononuclear macrophages (PBMCs) were isolated from the peripheral venous blood of septic patients (*n* = 7) and healthy controls (*n* = 8). **A** Western blot analysis of the indicated protein expression of KLF14 and IL-1β in PBMC samples from septic patients and healthy controls. **B** q-PCR analysis of KLF14 and IL-1β mRNA expression in PBMCs from septic patients and healthy controls. The data are presented as the mean ± SD. *P < 0.05, ***P < 0.001. **C**, **D** KLF14 was highly expressed in sepsis compared to healthy control samples in GEO data. The data are presented as the mean ± SD. **P < 0.01, ****P < 0.0001. **D** GSEA comparing “KLF14-low” versus “KLF14-high” sepsis patient blood samples revealed the most negatively enriched gene sets, including the “type II diabetes mellitus”, “drug metabolism”, “starch and sucrose metabolism”, and “glycan biosynthesis” gene sets

To determine whether KLF14 is altered in patients who suffer from sepsis and has clinical significance, we collected PBMCs from sepsis patients and control subjects. Compared with the respective levels in the control group, the mRNA and protein expression levels of KLF14 and the inflammatory factor IL-1β were increased in the PBMCs collected from sepsis patients (Fig. 6A, B). We further analyzed the biological information of patients with sepsis in the GEO database, and the results showed that the expression of KLF14 in sepsis patients was significantly higher than that in the control group (Fig. 6C). We also found that KLF14 was mainly enriched in pathways related to type 2 diabetes and sucrose signaling by gene set enrichment analysis (GSEA) of samples from septic patients with upregulated expression of KLF14 (Fig. 6D), revealing a correlation between KLF14 and glucose metabolism pathways. These findings further support the potential regulatory effects of KLF14 in human sepsis.

### Perhexiline, an agonist of KLF14, can significantly reduce the inflammatory state of endotoxemic and septic mice

**Fig. 7:**
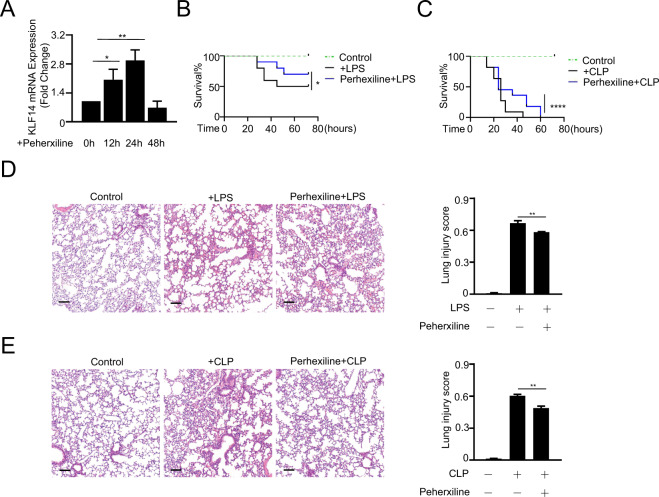
Perhexiline, an agonist of KLF14, can significantly reduce the inflammatory state of endotoxemic and septic mice. **A** The mRNA expression of KLF14 in lung tissues of WT mice after treatment with perhexiline (10 mg/kg, once per 12 h, twice, intragastric administration). **B**, **C** Perhexiline increased the survival rate of mice with LPS (20 mg/kg)-induced endotoxemia and CLP (25-gauge needle)-induced sepsis (the data are presented as the mean ± SD, *n* = 15, mice per group; *P < 0.05, ****P < 0.0001, Kaplan–Meier survival analysis). **D**, **E** Lung tissue of mice with CLP-induced sepsis and LPS-induced endotoxemia was assayed by hematoxylin and eosin staining (12 h; scale bars, 100 µm). The data are presented as the mean ± SD. *n* = 3, **P < 0.01

Previous results suggest that KLF14 may play a clinically significant role in human sepsis. Furthermore, studies have found that the cardiovascular drug perhexiline can induce the expression of KLF14 and increase blood HDL-C levels and cholesterol outflow ability by upregulating ApoA1 and ameliorating the development of atherosclerosis by targeting the KLF14 pathway [17]. Therefore, perhexiline can alleviate the inflammatory state by upregulating the expression of KLF14. Of note, our results indicate that perhexiline can induce the mRNA expression of KLF14 in the lung tissue of mice (Fig. 7A) and significantly prolong the survival of mice with LPS-induced endotoxemia and mice with CLP-induced sepsis (Fig. 7B, C). Moreover, perhexiline reduced the degree of lung tissue injury in both endotoxemic and septic mice (Fig. 7D, E). Taken together, these results indicated that perhexiline, as an agonist of KLF14, has a potential role in the management of sepsis.

## Discussion

As the first line of host defense, macrophages play a role in the pathophysiological process of sepsis. The function of macrophages is crucial for the prognosis of septic patients, and the metabolic state of macrophages can directly affect their immune function [18]. However, the mechanism underlying this process is still unknown. Here, our results identify KLF14 as a critical mediator that can affect the immune function of macrophages that is essential to the survival of septic mice. We provide evidence to support that KLF14 can inhibit glycolysis in macrophages by suppressing HK2 transcription during sepsis. In addition, pharmacological activation of KLF14 confers protection against sepsis in mice, supporting the therapeutic potential of KLF14 agonists in the treatment of sepsis.

Krüppel-like factors are associated with several aspects of leukocyte biology, including the regulation of T cells, monocytes/macrophages, and B cells [7]. Recently, publications have shown that the transcription factor KLF14 can regulate the differentiation of Treg cells by binding to the Treg-specific demethylation region (TSDR) enhancer region, and the deletion of KLF14 was found to alleviate the inflammatory response in colitis induced by DSS [12]. Other studies have shown that the upregulated expression of KLF14 protects the liver from immune-mediated damage by inducing Treg differentiation in autoimmune hepatitis (AIH) [19]. These seemingly contradictory results indicate that KLF14 is involved in the regulation of the immune response but also has different regulatory functions in different disease models. Sepsis involves a severe inflammatory response, the balance between systemic inflammatory response syndrome (SIRS) and the compensatory anti-inflammatory response (CARS) is crucial for prognosis, and macrophages are key players in the regulation of proinflammatory and anti-inflammatory responses. Therefore, the maintenance of macrophage immune function is of great importance for treating septic patients [20]. Interestingly, Wang et al. reported that the deletion of KLF14 can increase the inflammatory level of peritoneal macrophages, affecting the function of macrophages through NF-κB pathways in the regulation of atherosclerosis [21]. Similarly, in our experiments, we found that the deletion of KLF14 aggravated the inflammatory response in septic mice and that the severity of lung injury and systemic inflammation in septic mice was markedly higher than that in wild-type mice. In addition, by assessing KLF14^−/−^ BMDMs in vitro, we found that KLF14 could inhibit the secretion of inflammatory cytokines from macrophages. Notably, Wang et al. showed that the expression of KLF14 in THP-1-derived macrophages was decreased after LPS (100 ng/ml) stimulation for 4 h, while our results showed that KLF14 expression was increased after LPS stimulation for 6–24 h. This discrepancy suggests that KLF14 is a transcription factor that is widely involved in regulation and that the expression of KLF14 is likely to decrease and then increase in LPS-induced inflammatory models, but further experiments are needed to clarify the specific mechanism.

It has been reported that the metabolic dysfunction of leukocytes is a factor underlying the impaired function of the immune system of septic patients [22]. The regulation of glycolysis can directly affect the activation and immune function of macrophages, and glycolysis is specific to the M1 phenotype of macrophage polarization. While M2 macrophages rely on oxidative phosphorylation, metabolic dysfunction can affect the transformation from the M1 to M2 phenotype [23]. Of note, studies have shown that KLF14 can significantly regulate the metabolism of glucose and lipids [17] in cases of type 2 diabetes mellitus (T2DM) and reduced insulin sensitivity [24]. Moreover, recently reported findings revealed that the transcription factor KLF14 participates in antineoplastic effects during the development of colorectal cancer (CRC) by regulating the glycolytic enzyme LDHB, which confirmed the regulatory effect of KLF14 on glycolysis [13]. These studies indicate that KLF14 is involved in glucose metabolism; therefore, KLF14 may also affect glycolysis in macrophages. For further investigation, we investigated the effect of KLF14 on the function of macrophages and found that KLF14 mainly affects the glycolytic function of macrophages. Subsequently, we showed that KLF14 most likely affects hexokinase (HK2) by directly binding to the HK2 promoter to inhibit its transcription, thus affecting glycolysis. Interestingly, Small et al. identified 385 transgenes as potential targets of KLF14 but did not identify HK2. Therefore, we think the regulatory mechanism of KLF14 may be different in different cell lines [25].

The transcriptional regulatory function of KLF14 has been extensively reported, as KLF14 can activate the oxidative stress response by binding to the promoter of PLK1 (polo-like kinase 1), which is involved in endothelial cell regulation of T2D [26]. Here, we fully verified our hypothesis through rescue assays and ultimately identified the role of KLF14 in macrophage glycolysis during sepsis. We found that KLF14 can directly affect macrophages through the glycolytic enzyme HK2. Indeed, KLF14 may also restrain macrophages from secreting inflammatory factors through the NF-κB pathway during sepsis. However, we did not examine the impact of the NF-κB pathway on the regulatory role of KLF14. Existing studies have confirmed that KLF14 is a widely regulated transcription factor [11]. Therefore, we believe that there are indeed multiple regulatory pathways by which KLF14 affects macrophage functions. However, our study focused on the glycolysis-related effects of KLF14 on macrophage function, and other mechanisms of KLF14 warrant further investigation. Moreover, KLF14 is a maternally expressed gene [25], and it has been reported that there are significant sex differences in the prognosis of sepsis patients. The mortality rate of female sepsis patients is lower than that of male patients, which is likely due to the higher estrogen levels in female patients, which are protective [27]. Although we established female murine sepsis models, our results did not determine whether KLF14 is affected by sex differences, and further experiments are needed.

Although many studies have focused on the treatment of human sepsis in recent years, the heterogeneity of patients with sepsis hinders advances in this field [28]. To determine whether KLF14 has clinical significance in septic patients, we checked the expression of KLF14 in sepsis patients. The results showed that the expression of KLF14 in sepsis patients was significantly higher than that in the control group, and GEO data indicated that KLF14 was mainly enriched in type 2 diabetes and sucrose signaling pathways, identifying a correlation between KLF14 and glucose metabolism pathways. However, several studies have confirmed the regulatory role of KLF14 in glucose metabolism and immune-related diseases, but there is a lack of research on the clinical application of KLF14-targeted therapy. Previous studies have found that perhexiline can reduce the formation of atherosclerosis through activation of KLF14 expression, and this strategy is currently used in clinical cardiovascular-related diseases [17]. Here, our results showed that perhexiline can markedly improve the survival rate of endotoxemic and septic mice and reduce the inflammatory response of macrophages. However, Guo Yanhong et al. reported that perhexiline also increases HDL expression, and we wondered whether HDL can affect sepsis. We noticed that the level of HDL was increased in mice after treatment with perhexiline for 7 days in that study, and we believe that the increase in HDL levels occurred over a relatively long time. However, our results showed that KLF14 expression was altered after treatment with perhexiline for only 12 h. Therefore, we believe that the protective effect of perhexiline in sepsis may depend more on the influence of certain genes that are activated rapidly, such as KLF14. Due to the complexity of the relevant molecular functions, we cannot exclude the possibility that perhexiline may protect against sepsis by modulating HDL levels, and further research is needed. Although the underlying molecular mechanism of the KLF14 agonist perhexiline in sepsis was not elucidated in our study, we believe that KLF14 is a potential new therapeutic target for human sepsis, and related clinical trials are warranted.

**Fig. 8:**
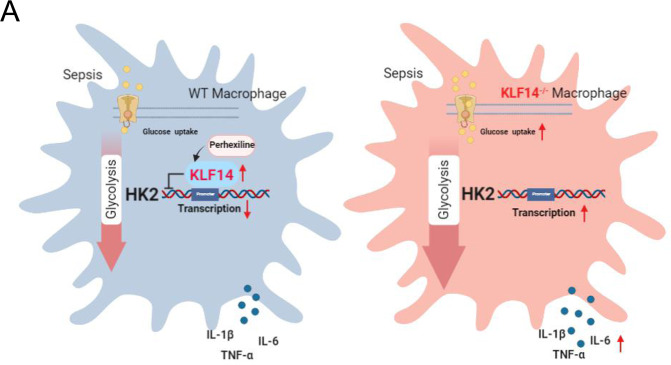
KLF14 regulates glycolysis and the immune function of macrophages by inhibiting the transcription of HK2 and may play a role in sepsis. Based on the previous findings, KLF14 is upregulated in sepsis and serves as an inflammation suppressor. KLF14 deletion promoted glycolysis in and inflammatory factor secretion by macrophages in sepsis. KLF14 regulated glycolysis in macrophages by inhibiting the transcription of HK2. Moreover, perhexiline activated the expression of KLF14 to reduce the immune response of macrophages upon LPS stimulation

In summary, our findings demonstrate that KLF14 plays an yet unrecognized role in the immune function of macrophages in sepsis, and thus, targeting KLF14 is a potential therapeutic strategy for sepsis. The expression of the transcription factor KLF14 was upregulated in both in vitro and in vivo sepsis models, and the deletion of KLF14 significantly decreased the survival rate of septic mice. Our findings showed that KLF14 is regulated during the immune response of macrophages via inhibition of HK2 transcription. Finally, the KLF14 agonist perhexiline improved the survival rate of septic mice and reduced the inflammation level of macrophages. The effect of KLF14 on other immune cells in sepsis awaits further investigation (Fig. 8).

## Supplementary information

Supplementary table1

Supplementary Figure1

Supplementary Figure2

Supplementary Figure3

Supplementary Figure4

Supplementary Figure5

Supplementary Figure6

## References

[1] Singer M, Deutschman CS, Seymour CW, Shankar-Hari M, Annane D, Bauer M (2016). The third international consensus definitions for sepsis and septic shock (Sepsis-3). JAMA.

[2] Gotts Jeffrey E, Matthay Michael A (2016). Sepsis: pathophysiology and clinical management. BMJ.

[3] Zeng L, Kang R, Zhu S, Wang X, Cao L, Wang H (2017). ALK is a therapeutic target for lethal sepsis. Sci Transl Med.

[4] Cheng Z, Abrams ST, Toh J, Wang SS, Wang Z, Yu Q (2020). The critical roles and mechanisms of immune cell death in sepsis. Front Immunol.

[5] Minhas PS, Latif-Hernandez A, McReynolds MR, Durairaj AS, Wang Q, Rubin A (2021). Restoring metabolism of myeloid cells reverses cognitive decline in ageing. Nature.

[6] Ganeshan K, Chawla A (2014). Metabolic regulation of immune responses. Annu Rev Immunol.

[7] Hsieh PN, Fan L, Sweet DR, Jain MK (2019). The Krüppel-like factors and control of energy homeostasis. Endocr Rev.

[8] Presnell Jason S, Schnitzler Christine E, Browne William E (2015). KLF/SP transcription factor family evolution: expansion, diversification, and innovation in eukaryotes. Genome Biol Evol.

[9] Chang E, Nayak L, Jain Mukesh K (2017). Krüppel-like factors in endothelial cell biology. Curr Opin Hemato.

[10] Chen X, Shi W, Zhang H (2020). The role of KLF14 in multiple disease processes. Biofactors.

[11] Yang Q, Civelek M (2020). Transcription factor KLF14 and metabolic syndrome. Front Cardiovasc Med.

[12] Sarmento Olga F, Svingen Phyllis A, Xiong Y, Xavier RJ, McGovern D, Smyrk TC (2015). A novel role for KLF14 in T regulatory cell differentiation. Cell Mol Gastroenterol Hepatol.

[13] Wu G, Yuan S, Chen Z, Chen G, Fan Q, Dong H (2019). The KLF14 transcription factor regulates glycolysis by downregulating LDHB in colorectal cancer. Int J Biol Sci.

[14] Hu W, Lu H, Zhang J, Fan Y, Chang Z, Liang W (2018). Krüppel-like factor 14, a coronary artery disease associated transcription factor, inhibits endothelial inflammation via NF-κB signaling pathway. Atherosclerosis.

[15] Fan G, Sun L, Shan P, Zhang X, Huan J, Zhang X (2015). Loss of KLF14 triggers centrosome amplification and tumorigenesis. Nat Commun.

[16] Yang M, Ren Y, Lin Z, Tang C, Jia Y, Lai Y (2015). Krüppel-like factor 14 increases insulin sensitivity through activation of PI3K/Akt signal pathway. Cell Signal.

[17] Guo Y, Fan Y, Zhang, Lomberk GA, Zhou Z, Sun L (2015). Perhexiline activates KLF14 and reduces atherosclerosis by modulating ApoA-I production. J Clin Invest.

[18] Lauterbach MA, Hanke JE, Serefidou M, Mangan M, Kolbe CC, Hess T (2019). Toll-like receptor signaling rewires macrophage metabolism and promotes histone acetylation via ATP-citrate lyase. Immunity.

[19] Chen X, Tan Q, Wang Y, Lv H, Wang Z, Lin Z (2019). Overexpression of KLF14 protects against immune-mediated hepatic injury in mice. Lab Invest.

[20] Rubio I, Osuchowski MF, Shankar-Hari M, Skirecki T, Winkler MS, Lachmann G (2019). Current gaps in sepsis immunology: new opportunities for translational research. Lancet Infect Dis.

[21] Wang H, Guo Y, Lu H, Luo Y, Hu W, Liang W, et al. Krüppel-like factor 14 deletion in myeloid cells accelerates atherosclerotic lesion development. Cardiovasc Res. 2021;cvab027. 10.1093/cvr/cvab027 PMC8803076 33538785

[22] Mcbride MA, Owen AM, Stothers CL, Hernandez A, Luan L, Burelbach KR (2020). The metabolic basis of immune dysfunction following sepsis and trauma. Front Immunol.

[23] Wang F, Zhang S, Vuckovic I, Jeon R, Lerman A, Folmes CD (2018). Glycolytic stimulation is not a requirement for M2 macrophage difffferentiation. Cell Metab.

[24] Voight BF, Scott LJ, Steinthorsdottir V, Steinthorsdottir V, Morris AP, Dina C (2010). Twelve type 2 diabetes susceptibility loci identified through large-scale association analysis. Nat Genet.

[25] Small KS, Hedman AK, Grundberg E, Nica AC, Thorleifsson G, Kong A (2011). Identification of an imprinted master trans regulator at the KLF14 locus related to multiple metabolic phenotypes. Nat Genet.

[26] Hao JS, Zhu CJ, Yan BY, Yan CY, Ling R (2018). Stimulation of KLF14/PLK1 pathway by thrombin signaling potentiates endothelial dysfunction in Type 2 diabetes mellitus. Biomed Pharmacother.

[27] Bösch F, Angele Martin K, Chaudry Irshad H (2018). Gender differences in trauma, shock and sepsis. Mil Med Res.

[28] Stanski Natalja L, Wong Hector R (2020). Prognostic and predictive enrichment in sepsis. Nat Rev Nephrol.

