# Supplementary material for: The transcription factor KLF14 regulates macrophage glycolysis and immune function by inhibiting HK2 in sepsis
Source: Cell Mol Immunol. 2022 Jan 4;19(4):504–15. doi: 10.1038/s41423-021-00806-5 (PMC8976055; doi:10.1038/s41423-021-00806-5)
Supplement: Supplementary file 1 — Supplementary table1 [file 41423_2021_806_MOESM1_ESM.doc]

**Supplementary Table 1. Clinical characteristics of non-septic patients and sepsis patients**

|  | **Control group (n=7)** | **Sepsis group (n=8)** |
| --- | --- | --- |
| **Age, mean (SD)** | 36.3 (5.6) | 61.3 (8.7) |
| **Gender n%**  **Male**  **Female** | 4 (57)  3 (43) | 4 (50)  4 (50) |
| **APACHEⅡ, mean (SD)** | N/A | 16.8 (10.5) |
| **SOFA, mean (SD)** | N/A | 7.9 (6.5) |
| **GCS, mean (SD)** | N/A | 12.5 (4.2) |
| **Length of stay, days, median (range)** | N/A | 17.25 (16.5) |
