# Supplementary material for: The transcription factor KLF14 regulates macrophage glycolysis and immune function by inhibiting HK2 in sepsis
Source: Cell Mol Immunol. 2022 Jan 4;19(4):504–15. doi: 10.1038/s41423-021-00806-5 (PMC8976055; doi:10.1038/s41423-021-00806-5)
Supplement: Supplementary file 2 — Supplementary Figure1 [file 41423_2021_806_MOESM2_ESM.pdf]

Supplementary Figure1

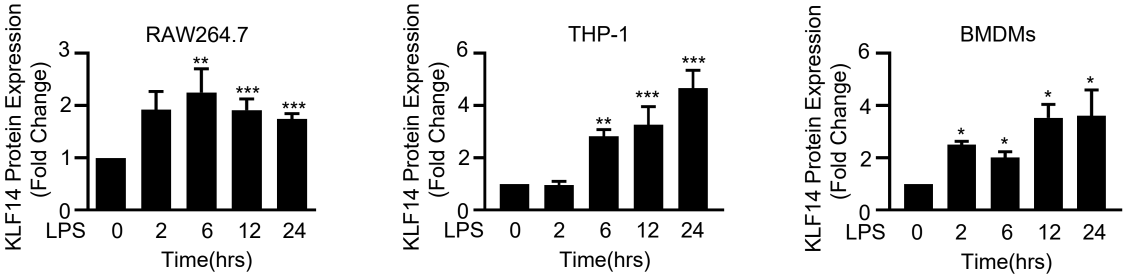

Supplementary Figure1. KLF14 is up-regulated in the macrophages under the stimulation of LPS. Quantification of the expression of KLF14 protein in RAW264.7, THP-1 derived macrophages and BMDMs under the LPS stimulation (100 ng/ml). Data are mean ± SD, n = 3, *P < 0.05, **P< 0.01, ***P < 0.001.
