# Supplementary material for: The transcription factor KLF14 regulates macrophage glycolysis and immune function by inhibiting HK2 in sepsis
Source: Cell Mol Immunol. 2022 Jan 4;19(4):504–15. doi: 10.1038/s41423-021-00806-5 (PMC8976055; doi:10.1038/s41423-021-00806-5)
Supplement: Supplementary file 3 — Supplementary Figure2 [file 41423_2021_806_MOESM3_ESM.pdf]

Supplementary Figure2

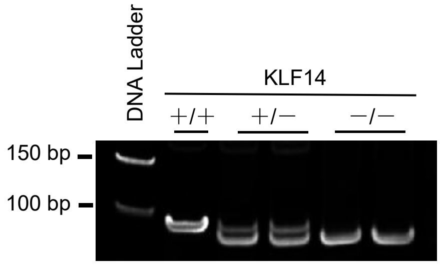

Supplementary Figure2. The gene identification of KLF14 knockout mice. Tissues were isolated from WT and KLF14-KO mice. One upper band is WT mice (+/+); two bands is heterozygous mice (+/−); one lower band is KLF14 knockout mice (−/−).
