# Supplementary material for: The transcription factor KLF14 regulates macrophage glycolysis and immune function by inhibiting HK2 in sepsis
Source: Cell Mol Immunol. 2022 Jan 4;19(4):504–15. doi: 10.1038/s41423-021-00806-5 (PMC8976055; doi:10.1038/s41423-021-00806-5)
Supplement: Supplementary file 4 — Supplementary Figure3 [file 41423_2021_806_MOESM4_ESM.pdf]

Supplementary Figure3

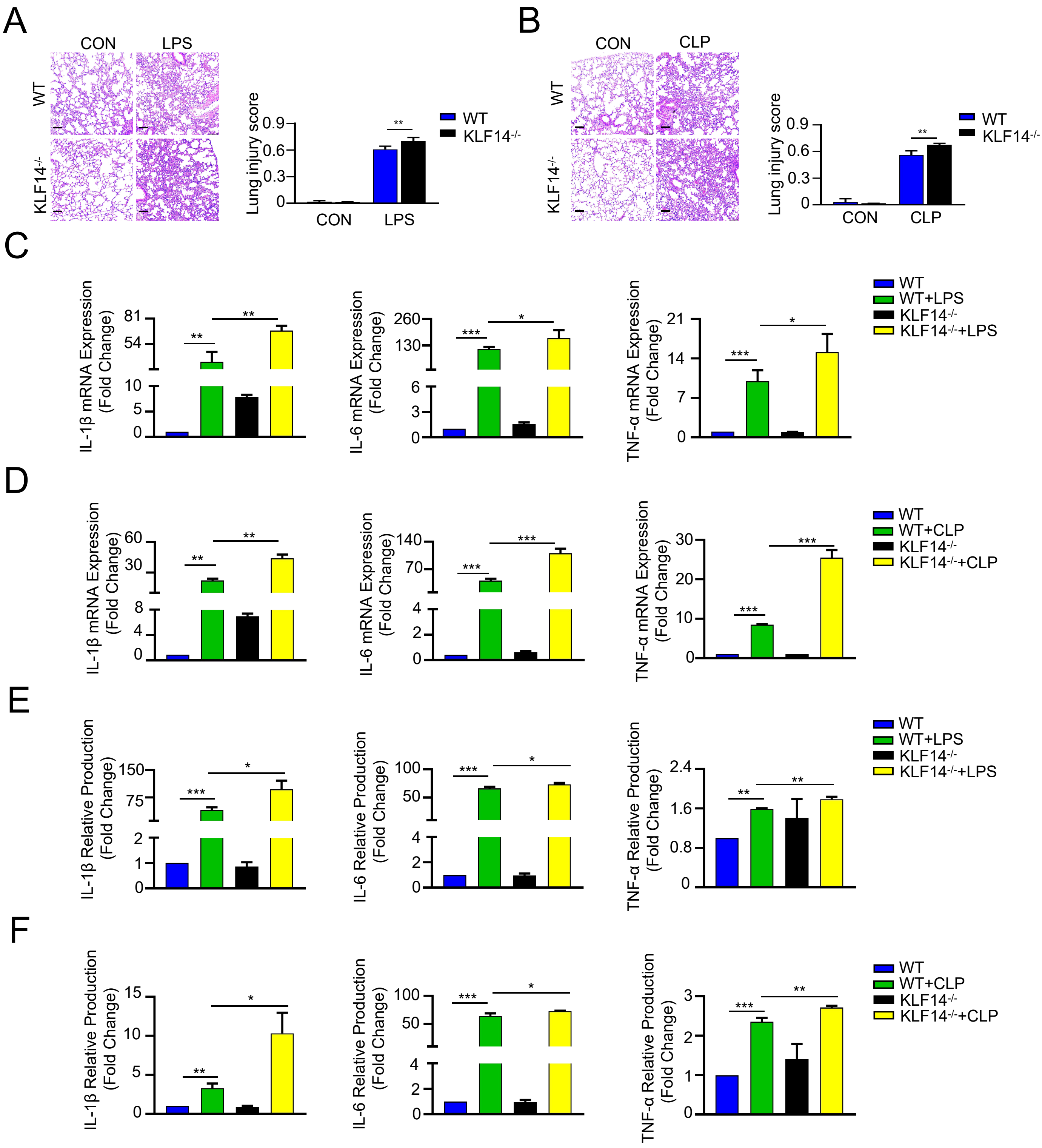

Supplementary Figure3. The deletion of KLF14 can significantly induce severe inflammation in female murine endotoxemia and sepsis models. Female wild-type and $KLF14^{-/-}$ knockout mouse were used for the construction of LPS-induced murine endotoxemia and CLP-induced murine sepsis models; (A-B) lung tissues hematoxylin and eosin staining (12h; scale bars, 100μm), (C-D) q-PCR analysis of inflammation cytokines of lung tissues and (E-F) ELISA analysis of cytokines isolated from peripheral blood were performend. (Data are mean ± SD, n = 3, *P < 0.05, **P< 0.01, ***P < 0.001)
