# Supplementary material for: The transcription factor KLF14 regulates macrophage glycolysis and immune function by inhibiting HK2 in sepsis
Source: Cell Mol Immunol. 2022 Jan 4;19(4):504–15. doi: 10.1038/s41423-021-00806-5 (PMC8976055; doi:10.1038/s41423-021-00806-5)
Supplement: Supplementary file 5 — Supplementary Figure4 [file 41423_2021_806_MOESM5_ESM.pdf]

Supplementary Figure4

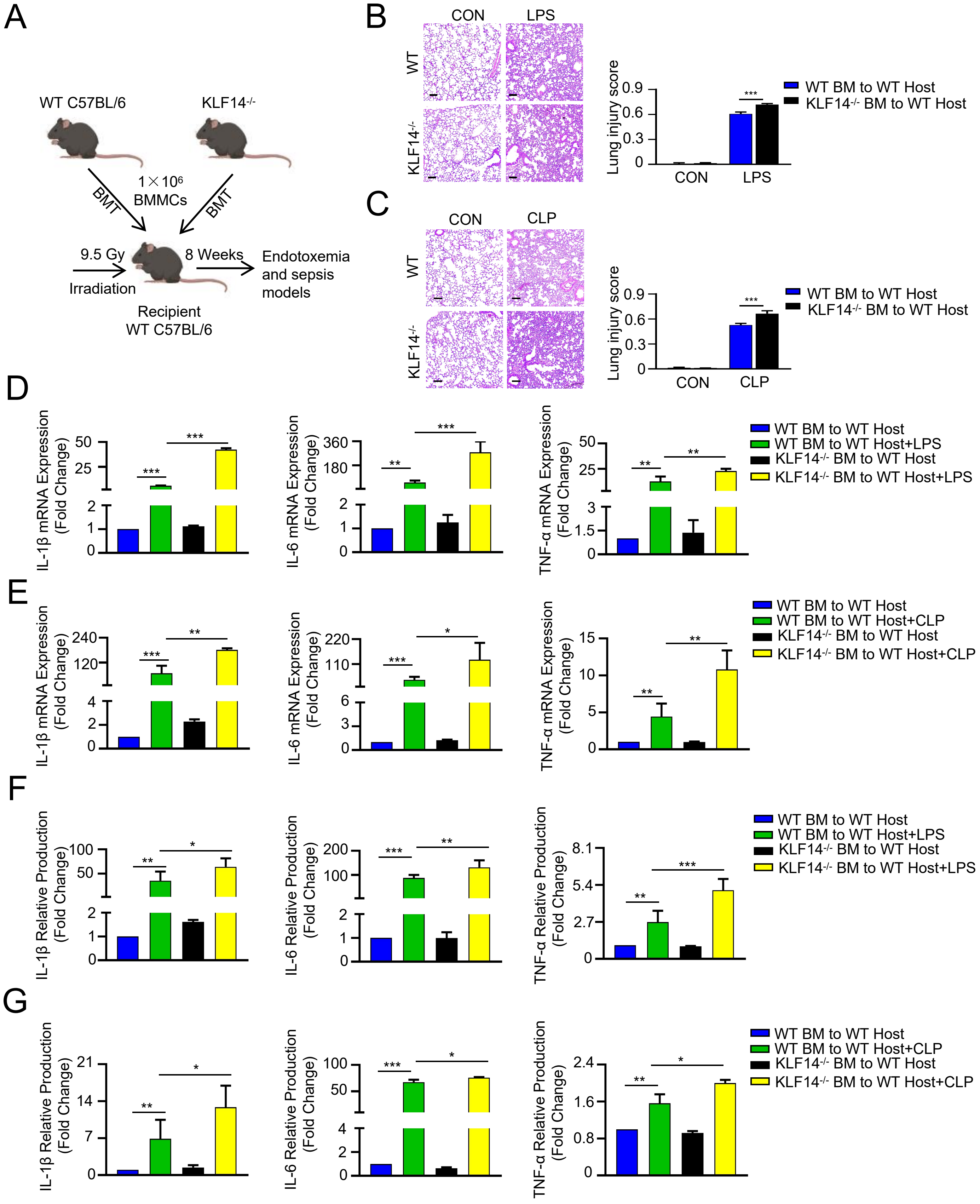

Supplementary Figure4. The deletion of KLF14 in bone marrow cells results in severe inflammation in murine endotoxemia and sepsis models. (A) The schematic of the bone marrow transplant experiments. Bone marrow cells from KLF14$^{-/-}$ mice and WT littermate controls were transplanted into WT C57BL/6 recipients. (B-G) After 8 weeks of transplantation, LPS-induced murine endotoxemia and CLP-induced murine sepsis models were construction; (B-C) lung tissues hematoxylin and eosin staining (12h; scale bars, 100μm), (D-E) q-PCR analysis of inflammation cytokines of lung tissues and (F-G) ELISA analysis of cytokines isolated from peripheral blood were performend. (Data are mean ± SD, n = 3, *P < 0.05, **P< 0.01, ***P < 0.001)
