# Supplementary material for: The transcription factor KLF14 regulates macrophage glycolysis and immune function by inhibiting HK2 in sepsis
Source: Cell Mol Immunol. 2022 Jan 4;19(4):504–15. doi: 10.1038/s41423-021-00806-5 (PMC8976055; doi:10.1038/s41423-021-00806-5)
Supplement: Supplementary file 6 — Supplementary Figure5 [file 41423_2021_806_MOESM6_ESM.pdf]

Supplementary Figure5

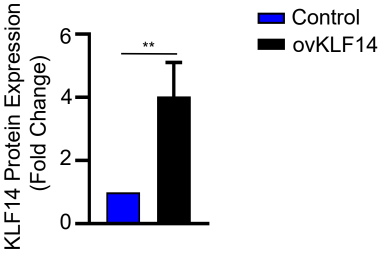

Supplementary Figure5. Quantification of KLF14 protein expression. Quantification of KLF14 protein expression in RAW264.7 macrophages identified the over-expressed of KLF14. (Data are mean ± SD, n = 3, **P< 0.01)
