# Supplementary material for: The transcription factor KLF14 regulates macrophage glycolysis and immune function by inhibiting HK2 in sepsis
Source: Cell Mol Immunol. 2022 Jan 4;19(4):504–15. doi: 10.1038/s41423-021-00806-5 (PMC8976055; doi:10.1038/s41423-021-00806-5)
Supplement: Supplementary file 7 — Supplementary Figure6 [file 41423_2021_806_MOESM7_ESM.pdf]

Supplementary Figure6

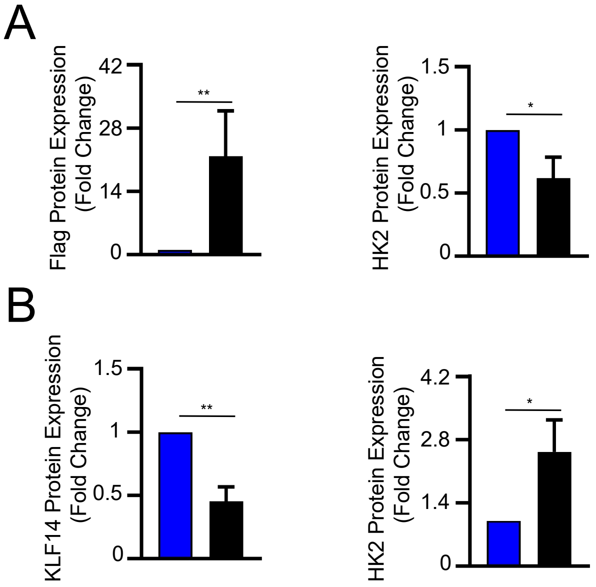

Supplementary Figure6. KLF14 inhibits the expression of Hexokinase 2. (A) Quantification of Flag and HK2 protein expression after transfected with KLF14-Flag plasmids in 293T cells; (B) Quantification of KLF14 and HK2 protein expression afte transfected with KLF14 siRNA in 293T cells. (Data are mean ± SD, n = 3, *P < 0.05, **P< 0.01)
